# Social Difficulties As Risk and Maintaining Factors in Anorexia Nervosa: A Mixed-Method Investigation

**DOI:** 10.3389/fpsyt.2018.00012

**Published:** 2018-02-26

**Authors:** Valentina Cardi, Núria Mallorqui-Bague, Gaia Albano, Alessio Maria Monteleone, Fernando Fernandez-Aranda, Janet Treasure

**Affiliations:** ^1^Section of Eating Disorders, Department of Psychological Medicine, Institute of Psychiatry, Psychology and Neuroscience, King’s College London, London, United Kingdom; ^2^Department of Psychiatry, University Hospital of Bellvitge-IDIBELL, Barcelona, Spain; ^3^CIBER Fisiopatología Obesidad y Nutrición (CIBEROBN), Instituto Salud Carlos III, Barcelona, Spain; ^4^Department of Psychology and Educational Sciences, University of Palermo, Palermo, Italy; ^5^Department of Psychiatry, University of Campania Luigi Vanvitelli, Naples, Italy

**Keywords:** anorexia nervosa, burdensomeness, fear of negative evaluation, submissiveness, social

## Abstract

Anorexia nervosa (AN) is a serious psychiatric disorder characterized by severe restriction of energy intake and dangerously low body weight. Other domains of functioning are affected, including social functioning. Although difficulties within this domain have started to be acknowledged by the literature, some important gaps remain to be filled. Do social difficulties predate the onset of the illness? What difficulties in particular are relevant for the development and maintenance of the illness? The aim of this study is to combine the use of quantitative and qualitative methods to answer these questions. Ninety participants with lifetime AN (88 women and 2 men) completed an online survey assessing memories of involuntary submissiveness within the family, fear of negative evaluation from others, perceived lack of social competence, feelings of social belonging, eating disorder symptoms, and work and social adjustment. Participants also answered three open questions regarding their experience of social relationships before and after the illness onset. The findings provided support for the hypothesized relationships between the study variables. Involuntary submissiveness and fear of negative evaluation predicted eating disorder symptoms and these associations were partially mediated by perceived lack of social competence. Two-thirds of the sample recalled early social difficulties before illness onset and recognized that these had played a role in the development of the illness. A larger proportion of participants stated that the eating disorder had affected their social relationships in a negative way. This study sheds some light on patients’ perspective on the predisposing and maintaining role that social difficulties play in AN and identifies key psychological variables that could be targeted in treatment.

## Introduction

Anorexia nervosa (AN) is diagnostically defined by abnormal eating behavior (i.e., restriction of energy intake followed by dangerously low body weight) and distorted attitudes toward body weight and shape (e.g., body dissatisfaction and exaggerated influence of physical appearance on self-evaluation) [DSM-5 (1)]. However, there are difficulties in other domains, such as social functioning. In the acute phase of the illness, patients often report isolation and loneliness (2, 3). In addition, they recall a preference to pursue solitary activities in childhood (4) and difficulties interacting with peers (5, 6). Parental reports confirm the extent of these difficulties (7) and corroborate the evidence that impairments in interpersonal functioning are not merely a consequence of the illness.

It is possible that both genetic and environmental experiences contribute to problems in social functioning in AN. Temperamental traits of harm avoidance (8) and shyness [e.g., Ref. (9)] have been consistently identified, even by siblings (10). Adverse early interpersonal experiences, such as exposure to sudden death of a relative (11), poor communication and care within the family (12, 13), and critical comments about the self {particularly regarding weight, shape and eating [e.g., Ref. (14–16)]}, have also been found to increase the risk for the development of eating disorder symptoms in longitudinal studies.

Interpersonal difficulties seem to predispose individuals to the onset of a number of psychiatric disorders [the so called “eco-phenotype” hypothesis (17)]. However, specific aspects, such as perceived involuntary submissiveness, high social and self-standards, and fear of negative evaluation may be of particular relevance to the development and maintenance of AN [e.g., Ref. (18–21)]. This is supported by patients’ tendency to feel inferior to others (22, 23), lack of assertiveness [e.g., Ref. (24)] and sensitivity to rejection (25), specific personality traits (26), and to specific personality traits and abnormal cognitive processing of social stimuli (27). Patients display attention biases toward threatening or rank-related stimuli, and negative interpretation biases of ambiguous scenarios depicting the risk of rejection [e.g., Ref. (22, 28, 29)]. They report lacking social skills and believe that they are a burden to close others as well as the rest of the society. Recent studies have found that perceived burdensomeness (PB) (i.e., lack of social competence) correlates with abnormal eating behaviors (30) and also with suicidal ideation (31, 32).

The primary aim of this paper is to use an experimental approach to identify potentially modifiable risk and maintaining factors of eating disorder psychopathology. The following hypotheses will be tested: (i) fear of negative evaluation (i.e., predisposing trait) and/or early experiences of involuntary submissiveness (i.e., environmental adversity) will predict eating disorder symptoms, and poor work and social adjustment and (ii) these relationships will be partially mediated by feelings of PB (mediator) in three separate models. A secondary aim is to conduct a qualitative analysis of patients’ answers to three open questions to establish the proportion of cases reporting social difficulties that predate the onset of the illness and their characteristics, the possible causal links between social difficulties and the eating disorder and the spectrum of poor social functioning after illness onset.

## Materials and Methods

### Participants and Procedure

Ninety-one participants (96.8% females) suffering from AN or subclinical AN (1) were recruited for the study. Eighty-nine participants completed the survey. All participants had been referred for outpatient treatment in England and had taken part in a large trial testing the use of online, guided self-help for AN between March 2014 and March 2016 (33, 34). This study tests hypotheses that are not related to those tested in the trial, and therefore, it reports original data. Participants were included if older than 18 years and excluded if suffering from a physical or mental illness requiring treatment in its own rights (i.e., diabetes mellitus, psychosis), as reported by their clinical teams. Patients were invited to complete an online assessment including sociodemographic questions and self-report measures. They were also asked to answer three open questions assessing the quality of their social life before and after illness onset and to comment on the possible role that early social difficulties have played in the development of the illness. The questions were: (i) “Please think back to the time when you were a child/adolescent, prior to the onset of your eating disorder. How would you describe your relationships with others at the time?” (ii) “Please think about your current situation. How do you experience the interaction with others now? How does your eating disorder affect your relationships at the moment?” and (iii) “Do you feel that the quality of your social relationships as a child/adolescent might have played a role in the development of your eating disorder? If so, in what way(s)?.”

All participants provided written signed informed consent and received monetary compensation for completing the survey. The study was approved by the Research Ethics Committee of London—Brent (14/LO/1347).

### Materials

The online survey consisted of the following standardised questionnaires:

#### Eating Disorder Examination Questionnaire (EDE-Q) (35)

A 36-item self-report questionnaire to assess attitudes and behaviors associated with eating disorders. All items are rated on a 0–6 Likert scale (with higher scores reflecting greater severity). The measure comprises a Global scale and four subscales (Restraint, Eating Concern, Weight Concern, and Shape Concern). In the present study, Cronbach’s alphas were as follows: restraint (α = 0.869), eating concern (α = 0.812), weight concern (α = 0.858), shape concern (α = 0.917), global score (α = 0.69).

#### Early Life Experiences Scale (ELES) (36)

The ELES measures memories of threat and submissiveness in childhood and consists of 15 items rated on a 5-point Likert scale (1 = “completely untrue” to 5 = “very true”). Scores are combined in a total score or three separate subscales: recall of feelings of threat (e.g., “I experienced my parents as powerful and overwhelming”), feeling (un)valued (e.g., “I felt very comfortable and relaxed around my parents”) and submissiveness (e.g., “I often had to give in to others at home”). Higher scores correspond to higher levels of threat, submissiveness and feeling (un)valued. In the present study Cronbach’s alphas for the different subscales were as follow: 0.60 for (un)valued, 0.89 for submissiveness and 0.90 for threats.

#### Brief Fear of Negative Evaluation (BFNE) (37)

The BFNE includes 12 items and measures fear of being negatively evaluated by others. It is a shorter version of the original Fear of Negative Evaluation questionnaire (38). Scores are expressed using a 5-point Likert scale ranging from 1 (“not at all”) to 5 (“extremely”). The removal of the four reversed scored items has been found to improve the validity of this scale (39) and therefore these items were removed from the analysis in this study. In the current sample, the questionnaire presented excellent internal consistency (α = 0.92).

#### Interpersonal Needs Questionnaire (INQ) (40)

The INQ assesses thwarted belongingness (TB) and PB. It consists of 15 items rated on a 7-point Likert scale and participants are requested to identify how true each items feels to them. The TB includes nine items and the PB subscale includes six items. Higher scores on each subscale indicate higher levels of perceived TB and burdensomeness. The validation study revealed that both of these constructs had convergent associations with interpersonal constructs, such as loneliness, social support and social worth, and were associated with suicidal ideation (40). The Cronbach’s alpha in this study was 0.915 for PD and 0.919 for TB.

#### The Work and Social Adjustment Scale (WSAS) (41)

The WSAS is a 5-item self-report scale designed to measure the degree of functional impairment. All items are rated on a 9-point Likert-type scale, ranging from 0 (no impairment) to 8 (very severe impairment). The maximum total score is 40, with higher scores representing greater impairment. The WSAS has demonstrated good internal consistency and test-retest reliability and is sensitive to patients’ perceptions of disorder severity (41). In the present study, internal consistency for the WSAS total score was α = 0.85.

### Data Analyses

All data were analysed using IBM SPSS Statistics 24.0 software (SPSS, Chicago, IL, USA). Relationships between each of the studied variables (i.e., EDE-Q, INQ-PB, BFNES-R, ELES, WSAS, DASS, EXITS) were assessed using Pearson product-moment correlation coefficients (*r*). Alpha was set at *p* < 0.05. After the correlation analysis, the Hayes’s PROCESS macro (42) was used to test Lambert’s mediation model (i.e., indirect effect) by estimating: (1) the effect of early memories of involuntary submissiveness on eating disorder symptoms through PB, (2) the effect of fear of negative evaluation on eating disorder symptoms through PB, and (3) the effect of fear of negative evaluation on work and social adjustment through PB.

The answers to the open questions were analysed using thematic analysis to identify, analyse and report the recurrent patterns produced in response to the questions [“themes” (43)]. The data analysis was undertaken manually and by using the qualitative data analysis software NVivo (version 11). GA and VC familiarised themselves with the data through repeated readings, identified the initial codes (i.e., sentence by sentence coding), and sorted the codes into themes and overarching themes. Themes were then revised for coherence and distinctiveness and were defined and named (43).

## Results

### Demographic and Clinical Characteristics

The final sample included 88 women and 2 men with a diagnosis of AN (*N* = 60) or atypical AN [*N* = 30, all diagnostic criteria were met, except for body mass index that was within the normal range, DSM-5 (1)]. Most of the patients were employed (91.4%). Half were single (50.6%) and 6.4% were separated or divorced. Thirty seven percent had had previous hospital admissions for an eating disorder and 48% were on psychiatric medication. Forty patients reported binge and purging episodes over the previous 4 weeks. Clinically significant levels of eating disorder symptoms (44–46), functional impairment (WSAS mean scores between 10 and 20) and fear of negative evaluation (BFNE mean scores above 25) were found. High levels of PB (i.e., lack of social competence) and TB were also reported. Table 1 displays means (and SDs) for the demographic, clinical and social functioning measures.

**Table 1:** Demographic, clinical and social functioning characteristics.

	Mean (SD)	Range
Age	28.9 (11.1)	18–63
Years of education	15.8 (3.0)	9–24
Body mass index	17.8 (1.9)	12.83–23.80
Duration of illness (years)	8.4 (10.4)	0–46
Age at illness onset	19.9 (8.8)	6–58
Age when illness diagnosed	24.0 (10.3)	12–62
Eating Disorder Examination Questionnaire (total score)	3.1 (1.5)	0–5.76
Work and Social Adjustment Scale	16.0 (9.3)	9–39
Brief fear of negative evaluation	32.3 (8.0)	2–40
Early Life Experiences Scale (ELES) (memories of involuntary submissiveness)	19.3 (6.6)	5–30
ELES (memories of threat)	13.0 (7.0)	6–30
ELES (memories of feeling valued)	4.7 (1.7)	1–9
Interpersonal Needs Questionnaire (INQ) (perceived burdensomeness)	19.5 (10.2)	6–42
INQ (thwarted belongingness)	27.6 (14.2)	5–62

*Data expressed as means (SDs), and ranges*.

### Correlation Analyses

Correlation analyses show significant associations between the studied variables. Specifically, the EDE-Q total score significantly correlated with ELES-Submissiveness, WSAS, BFNES and INQ-PB, with correlation indices ranging from 0.34 to 0.58. Also, INQ-Burden scores significantly correlated with ELES-Submissiveness, WSAS and BFNES with correlation indices ranging from 0.31 to 0.47 (see Table 2, for the correlation indices on the studied variables).

**Table 2:** Correlations between duration of illness, work and social adjustment (WSAS), eating disorder symptoms (EDE-Q total score), early memories of submissiveness (ELES-Submissiveness), fear of negative evaluation (BFNES) and perceived burdensomeness (INQ-PB).

	Illness duration	EDE-Q-total	ELES-Submissiveness	WSAS total	BFNES total
Duration of illness	–				
EDE-Q total	0.001	–			
ELES-Submissiveness	0.240*	0.340**	–		
WSAS	−0.019	0.579**	0.166	–	
BFNES	0.028	0.364**	0.180	0.311**	–
INQ-PB	−0.033	0.455**	0.314**	0.470**	0.388**

*p < 0.05, **p < 0.001.

### Mediation Models

**Figure 1:**
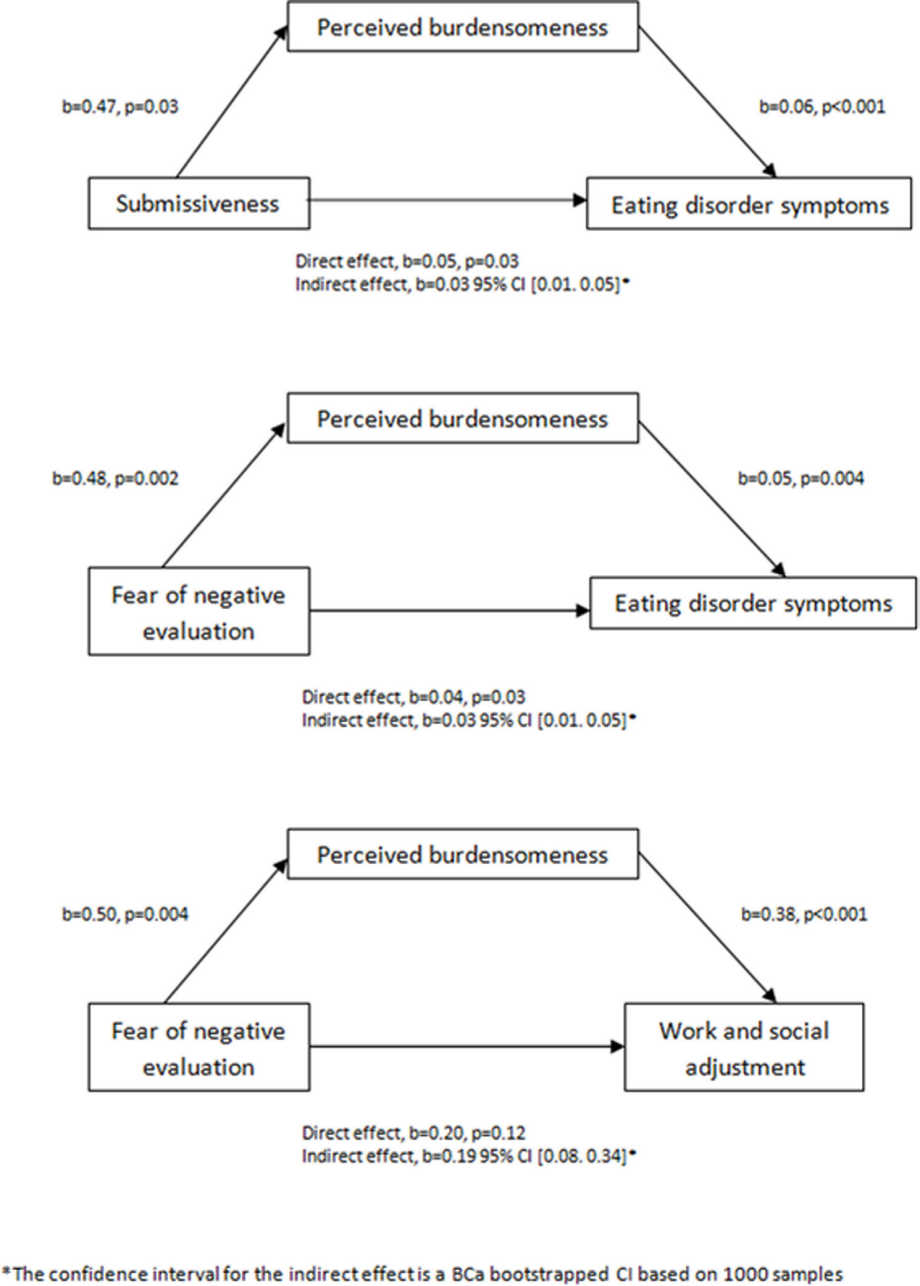
Three mediation models of early life experiences of submissiveness (ELES-Submissiveness) and fear of negative evaluation (BFNES) as predictors of eating disorder symptoms (EDE-Q) and of work and social adjustment (WSAS), mediated by perceived burdensomeness (INQ-PB).

The first model indicated that early life experiences of submissiveness (ELES-Submissiveness) were a significant predictor of PB (INQ-PB; *b* = 0.47, *t* = 3.01, *p* = 0.03) and that burdensomeness was a significant predictor of eating disorder symptoms (EDE-Q; *b* = 0.06, *t* = 3.95, *p* < 0.001). Memories of submissiveness significantly predicted eating disorder symptoms both when burdensomeness was (*b* = 0.05, *t* = 2.25, *p* < 0.03; *R*^2^ = 0.25) and was not included (*b* = 0.08, *t* = 3.37, *p* < 0.001; *R*^2^ = 0.11) in the model. Finally, there was a significant indirect effect of recalled submissiveness on eating disorder symptoms through PB [*b* = 0.03, BCa CI (0.01, 0.05)] (see Figure 1). The second model indicated that fear of negative evaluation (BFNES) was a significant predictor of PB (INQ-PB; *b* = 0.48, *t* = 3.89, *p* < 0.001) and that PB was a significant predictor of eating disorder symptoms (EDE-Q; *b* = 0.05, *t* = 3.65, *p* < 0.001). Fear of negative evaluation significantly predicted eating disorder symptoms both when PB was (*b* = 0.04, *t* = 2.19, *p* = 0.03; *R*^2^ = 0.25) and was not included (*b* = 0.07, *t* = 3.65, *p* < 0.001; *R*^2^ = 0.13) in the model. Finally, there was a significant indirect effect of fear of negative evaluation on eating disorder symptoms through PB [*b* = 0.0128, BCa CI (0.01, 0.05)] (see Figure 1). The third model indicated that fear of negative evaluation (BFNES) was a significant predictor of PB (INQ-PB; *b* = 0.50, *t* = 3.70, *p* < 0.001) and that PB was a significant predictor of work and social adjustment (WSAS; *b* = 0.38, *t* = 4.05, *p* < 0.001). Fear of negative evaluation significantly predicted work and social adjustment when PB was not in the model (*b* = 0.39, *t* = 3.03, *p* < 0.001; *R*^2^ = 0.96), but not when it was included in the model (*b* = 0.20, *t* = 1.55, *p* = 0.13; *R*^2^ = 0.24). Finally, there was a significant indirect effect of fear of negative evaluation on work and social adjustment through PB [*b* = 0.1918, BCa CI (0.08, 0.34)] (see Figure 1).

### Patients’ Narratives

Ninety participants replied to three open questions. The first question asked to think back to the time when they were a child/adolescent (prior to the onset of the eating disorder) and to describe their relationships with others at the time. Forty participants recalled important difficulties within the interpersonal domain and 22 participants reported interpersonal difficulties to a lesser degree; the remaining participants (*N* = 28) did not report early interpersonal difficulties (Table 3). The second question related to their current social situation and how their eating disorder impacted on their social life. Eighty-one participants reported that the eating disorder had had a negative impact on their social life. Only eight subjects reported that the eating disorder did not cause any change to their social relationships (Table 3). The last question asked participants whether they thought that the quality of their social relationships as child/adolescent might have played a role in the development of the eating disorder and if so, in what ways. Sixty-three participants responded that yes, they thought that early social difficulties might have played a role in the development of the illness, four people replied that they were not sure, and 23 reported that they could not see a link between their early social life and the eating disorder (Table 3). Table 3 presents the overview of the themes that emerged in relation to each question.

**Table 3:** Main themes emerging in response to three open questions to investigate the quality of interpersonal relationships before and after illness onset, and the potential role that early social difficulties played in the development of the illness.

Question	Answer	Main themes	Examples
Please think back to the time when you were a child/adolescent, prior to the onset of your ED. How would you describe your relationships with others at the time	Difficulties causing significant impairment of social functioning (*N* = 40)	Fear of rejection	*“Felt pressure to fit in with peers and be in the ‘popular’group. Worried about what others thought about me (including my looks) and wanted everyone to like me. Felt pressure to always try to ‘impress’ my dad for attention/him to notice me”*
		Feeling different from others	*“I generally found it difficult interacting with peers, feeling I had interests that others didn’t generally share”*
		Lack of social skills	*“Found very challenging/unable to join or interject in a conversation. It was very tiring and stimulating to be around people so I struggled with wanting to be with others while needing alone time”*
		Low self-esteem	*“I struggled to interact as had no confidence within myself, always worries people seen me as fat”*
		Being victim of bullism	*“Bullying was an issue in school. Not only from peers but from teachers when I was in primary school then it continued in secondary school. So interaction was a big problem for me. I felt isolated and alone throughout growing up”*
Responders: *N* = 90		Problems within family	*“Having been abused as a child I did not make friends easily and mistrusted everyone”*
		Comorbid disorder	*“I have recently been diagnosed with Asperger which retrospectively had a big impact on my relationships growing up. I never really understood my peers and the things they were interested in and as such didn’t have many close friends”*

	Some difficulties that did not cause impairment to social functioning (*N* = 22)	Difficulties to interact with strangers and within groups	*“I was always a little shy and socially awkward around people I don’t know but very comfortable around people I know”*
		Difficulties interacting with peers	*“I have always been quite naturally shy but could generally interact with others quite well, usually more comfortable in the presence of adults than of peers my age”*
	No difficulties recalled (*N* = 28)	Easy to interact with others	*“Before my eating disorder I had good social relations, I found it easy to interact with others, of all ages and backgrounds. I felt comfortable around adults, and was able to adapt to different situations”*

Please think about your current situation. How do you experience the interaction with others now? How does your ED affect your relationships at the moment?	The eating disorder had a negative impact on interpersonal relationships (*N* = 81)	Lack of flexibility in social situations	*“I think without an eating disorder I would be able to do more social things and be confident to be in different situations without anything being so planned”*
Responders: *N* = 89		Intense experience of negative feelings	*“I would like to be able to be free and happy and be able to attend social events without fear, worry, guilt and shame”*
		Lack of social skills	*“I much prefer ‘doing things’ with people—e.g., going for a walk, than just sitting down and talking as I get restless and don’t know what to talk about. I feel uncomfortable if there are pauses and don’t know what to say. I find it easier making small talk and talking to strangers than talking to people I know better”*
		Isolation due to fear of food or body image concerns	*“My relationships are very much affected by my eating disorder. It has isolated me from my family and friends and I don’t spend much time with them at all. This is mainly due to the fear of having to eat while with them/them seeing me eat, etc.”*
		Fear of being judged for having an eating disorder	*“I don’t what people to find out and think I’m a freak and stop interacting with me”*
		Poor capacity to concentrate on conversations due to eating disorder thoughts	*“I do find it hard when such events are based around food, as I find it hard to focus on chatting to people when I am aware of the presence of food (particularly buffet-style) nearby—my main focus is on eating the food as I consider it to be ‘allowed’ as ‘a treat’”*

	The eating disorder did not have any impact on interpersonal relationships (*N* = 8)	Social difficulties ascribed to comorbid mental disorders	*“If I didn’t have eating disorder it would still be the same because I have a social phobia”*

Do you feel that the quality of your social relationships as child/adolescent might have played a role in the development of your eating disorder? If so, in what way(s)?	The eating disorder is a way to respond to a need (*N* = 63)	Need to fit in	*“Yes, definitely! I wanted to be a popular, attractive girl, have many friends and a boyfriend. Getting skinny was supposed to be a way to do that”*
Responders: *N* = 90		Need to feel control	*“When I lost control of other things in my life (family related and later, stress related), I started restricting to cope and feel better”*
		Need to hide due to inadequacy or way to self-punish	*“I always felt like a bit of a sub-par person in terms of how others regarded me which contributed to the feelings like I do not deserve things like normal people do. Like food”*
		Need to distract from loneliness and isolation	*“I would often eat for comfort as an adolescent, consoling my lack of human connection with large amounts of fatty/sugary foods. This resultantly contributed to a move in the opposite direction, a hyper-awareness of that tendency and a connection between restriction and social ‘success’”*
		Restriction of energy intake as a mean of silencing negative thoughts and emotions	*“I believe I have always felt responsibility for other people’s happiness, which makes it difficult for me to express negative emotions. I fear abandonment and rejection. For this reason, I have tended to internalize negative thoughts and used food restriction as a means of managing them”*

	Not sure as to whether early social experiences have played a role in the eating disorder onset (*N* = 4)		*“The development of my eating disorder was mainly due to low self-esteem, but I don’t think that was a result of my social relationships. I grew up with a twin sister who helped me with social relationships”*
	Definitely no role played (*N* = 23)		*“Not really as I was always sociable and got to spend loads of time with my friends and family”*

*Extracts of participants’ answers are provided as examples*.

## Discussion

The aim of this paper was to test the hypotheses that: (i) fear of negative evaluation (i.e., predisposing trait) and early experiences of involuntary submissiveness (i.e., environmental adversity) would predict eating disorder symptoms and poor work and social adjustment, (ii) that this relationships would be partially mediated by feelings of PB (mediator) and (iii) that patients’ narratives would highlight that early social difficulties predate the onset of the illness and are causally linked to the development of the eating disorder; and that they would indicate that the eating disorder had had a negative impact on social functioning. The findings corroborate the first two hypotheses, in that involuntary submissiveness and fear of negative evaluation predicted eating disorder symptoms and these associations were partially mediated by perceived lack of social competence. Regarding the third hypothesis, approximately two-thirds of the participants in the study (*N* = 62/90) could recall early social difficulties before illness onset and an even greater proportion (81/90) recognised that the eating disorder had affected their social relationships in a negative way. When asked whether social difficulties had played a role in the development of the illness, two-thirds of the sample (*N* = 63) confirmed that this was the case.

The findings of this study add weight to the hypotheses that suggest that social difficulties are important in the maintenance of the eating disorder [e.g., Ref. (18–20, 24, 26)]. Importantly, these findings also demonstrate that social difficulties precede the onset of the disorder in a subgroup of patients. This is particularly relevant considering the paucity of studies investigating the chronology of the occurrence of social difficulties in AN (47). The identification of subgroups of individuals for whom social difficulties play a role in the onset of the disorder might inform the development of treatments that have greater specificity and relevance.

This study provides the first evidence that submissiveness and fear of negative evaluation in particular might be causally involved in the development of the eating disorder psychopathology. Compared to the general population, people with a lifetime diagnosis of AN have recalled memories of submissiveness within the family to a greater extent than the general population (36, 48). This provides support for Hilde Bruch’s (18) early theorisations of the illness that people with AN struggle with asserting themselves within the family and tend to act in response to others’ expectations instead (49). The finding that patients experience an intense fear of negative evaluations supports Bruch’s etiological hypothesis further and corroborates more recent conceptualisations of AN [e.g., Ref. (19, 24)]. It also extends the literature that have found relationships between fear of negative evaluation and abnormal eating attitudes in non-clinical samples [e.g., Ref. (50–52)], by adding evidence from a large clinical population. Evolutionary theories of the eating disorder psychopathology suggest that dietary restriction and weight loss could be described as responses to the threat of social exclusion, by attempting to become more vulnerable and attractive [e.g., Ref. (53, 54)] or to gain status and control (55).

Finally, this study indicates that PB mediates the relation between involuntary submissiveness and fear of negative evaluation, and eating disorder symptoms. The levels of burdensomeness reported in this study’s population were similar to those of patients with eating disorders who also reported lifetime suicidal attempts (32) and were higher than those reported by patients suffering from other psychiatric conditions (31). This is particularly relevant, considering that the sample in this study included people at different stages of the illness and recovery, and posits the need to address patient’s perception of social competence at all stages of treatment and in relapse prevention programs.

### Limitations

The main limitation of this study is its cross-sectional design. This means that findings should be interpreted cautiously and replicated using more appropriate experimental designs. The answers to the ELES and the open questions are based on retrospective recall of early experiences and might be subject to memory biases. The hypothesis that memories of adverse early experiences could be amplified or overrepresented in people experiencing current psychopathological symptoms is partially supported in patients with social anxiety [for review, see Ref. (56)]. The literature on memory bias in patients with AN suggests no biases for memories triggered by depression-related words including “rejection” and “criticism” (57) and no differences in terms of frequency of negatively valenced memories (57, 58) when patients are compared to healthy controls. However, there is some indication that patients with AN tend to recall fewer and more general (vs. specific) memories when these are cued by food- or body-related words (57). Taken together, these findings do not seem to suggest that negative interpersonal memories are easily biased in patients with eating disorders.

Only patients with a lifetime diagnosis of AN were included in this study, which does limit the conclusions that can be extended to other eating disorder diagnostic subgroups. The qualitative analyses were based on answers to open questions, rather than semi-structured interviews that would have allowed follow-up questions and more flexibility.

### Clinical Implications

Despite the limitations outlined above, this study identifies potentially modifiable predisposing and maintaining factors of AN that could be targeted in treatment. Three specific factors might be implicated in the onset and maintenance of AN: fear of negative evaluation, perceived lack of social competence and early experiences of submissiveness. Targeting processes specifically implicated in illness’ maintenance may improve the effectiveness of treatments (59). A number of treatments have now been adapted or developed to target interpersonal difficulties in eating disorders (e.g., interpersonal psychotherapy, cognitive analytic therapy, focal psychoanalytic therapy), with various degrees of specificity and success. The use of treatment adjuncts targeting isolated maintaining factors, such as “Cognitive Remediation and Emotion Skills training” to address emotion regulation difficulties and cognitive rigidity [e.g., Ref. (60)], and cognitive bias modification training to target cognitive biases to the threat of rejection (33, 34) might hold promise to improve treatment effectiveness in eating disorders.

## Conclusion

This study contributes to the literature that supports a link between social difficulties and eating behaviors by pointing to three specific factors (fear of negative evaluation, perceived lack of social competence and early experiences of submissiveness) that might predispose and/or maintain AN. The cross-sectional design of the study means that findings should be interpreted cautiously and replicated using a longitudinal design.

## Ethics Statement

The study was approved by the Research Ethics Committee of London—Brent (14/LO/1347).

## Author Contributions

VC contributed to the conception and design of the study, to data acquisition and analysis, and to data interpretation and manuscript preparation. NB contributed to data analysis and interpretation and to manuscript preparation. GA contributed to data collection and manuscript preparation. FA and AMM contributed to the interpretation of data and manuscript preparation. JT contributed to the conception and design of the study, to data interpretation and manuscript preparation. All the authors have approved the final version of the manuscript and the attached figures. All the authors have agreed to all aspects of the work.

## Conflict of Interest Statement

The authors declare that the research was conducted in the absence of any commercial or financial relationships that could be construed as a potential conflict of interest.
